# A Water-Based and Continuous-Flow-Usable Cascade: Sustainable Synthesis of Tetrazolo-Fused Heterocycles

**DOI:** 10.1021/jacsau.5c01089

**Published:** 2025-10-24

**Authors:** Yaxi Wang, Suxian Fu, Deshu Kong, Lu Hu, Siping Pang, Jean’ne M. Shreeve

**Affiliations:** † School of Materials Science & Engineering, 47833 Beijing Institute of Technology, Beijing 100081, China; ‡ Department of Chemistry, 5640 University of Idaho, Moscow, Idaho 83844-2343, United States

**Keywords:** continuous flow, energetic materials, water-based cascade, heterocycles, green chemistry

## Abstract

The innovation of energetic materials is frequently hampered by economic inefficiency and safety concerns. Emerging cascade reactions could overcome existing bottlenecks and accelerate next-generation energetic materials with enhanced scalability and reduced risks. In this work, a water-based cascade reaction has been applied to obtain 6-amino-7,8-dihydrotetrazolo­[5,1-*f*]­[1,2,4]­triazin-8-ol (**AHTO**), a new nitrogen-rich energetic compound. Using water as a green, multifunctional medium, this strategy achieves three critical advances: (1) elimination of intermediate isolation through cascade reaction integration, (2) minimization of toxic solvents and hazardous reagents, and (3) stabilization of sensitive tetrazolo intermediates. The inherent safety benefits of water-mediated chemistry are further amplified through microchannel continuous-flow technology, leading to an all-round improvement. Finally, **AHTO** was obtained in a 90.8% crystallization yield and a 98.1% HPLC purity in a 3 h flow reaction. After nitroamino derivatization, 6-nitroamino-7,8-dihydrotetrazolo­[5,1-*f*]­[1,2,4]­triazin-8-ol (**NHTO**) was prepared as a new potential metal-free primary explosive.The integration of cascade with flow chemistry will accelerate the development efficiency of energetic materials while balancing economic, safety, and environmental considerations.

## Graphical abstract

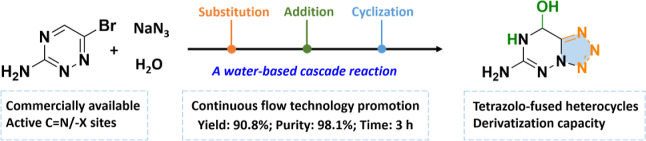

## 1 Introduction

Energetic materials have significantly impacted the course of human civilization. These special materials are the cornerstone of aerospace, underground exploration, and national defense.^1,2^ Since energetic materials are often associated with energy-safety compatibility issues, this places high performance demands on the energetic compounds in formulations.^3,4^ Over the development of nearly 150 years, each generation of classic energetic compounds is shown in Figure 1a. For example, 1,3,5-triamino-2,4,6-trinitrobenzene (TATB) has exceptional stability toward external stimuli.^5^ The energy level advances significantly from 1,3,5-trinitro-1,3,5-triazacyclohexane (RDX), to 1,3,5,7-tetranitro-1,3,5,7-tetrazocine (HMX), and to 2,4,6,8,10,12-hexaazaisowurtzitane (CL-20).^6,7^ However, there are still many common problems in actual industrialized production.^8^ For example, stepwise routes are always designed to cope with uncertainties in the risky energy climb.^9^ Overreliance on organic solvents and strong oxidative conditions can pose environmental and safety concerns.^10^ The cost of some synthetic steps (e.g., precious metal catalysts in the CL-20 synthesis) can also constrain practical applications.^11^ Thus, it is crucial to develop innovative strategies to achieve the sustainable development of new energetic compounds.^12,13^

**Figure 1:**
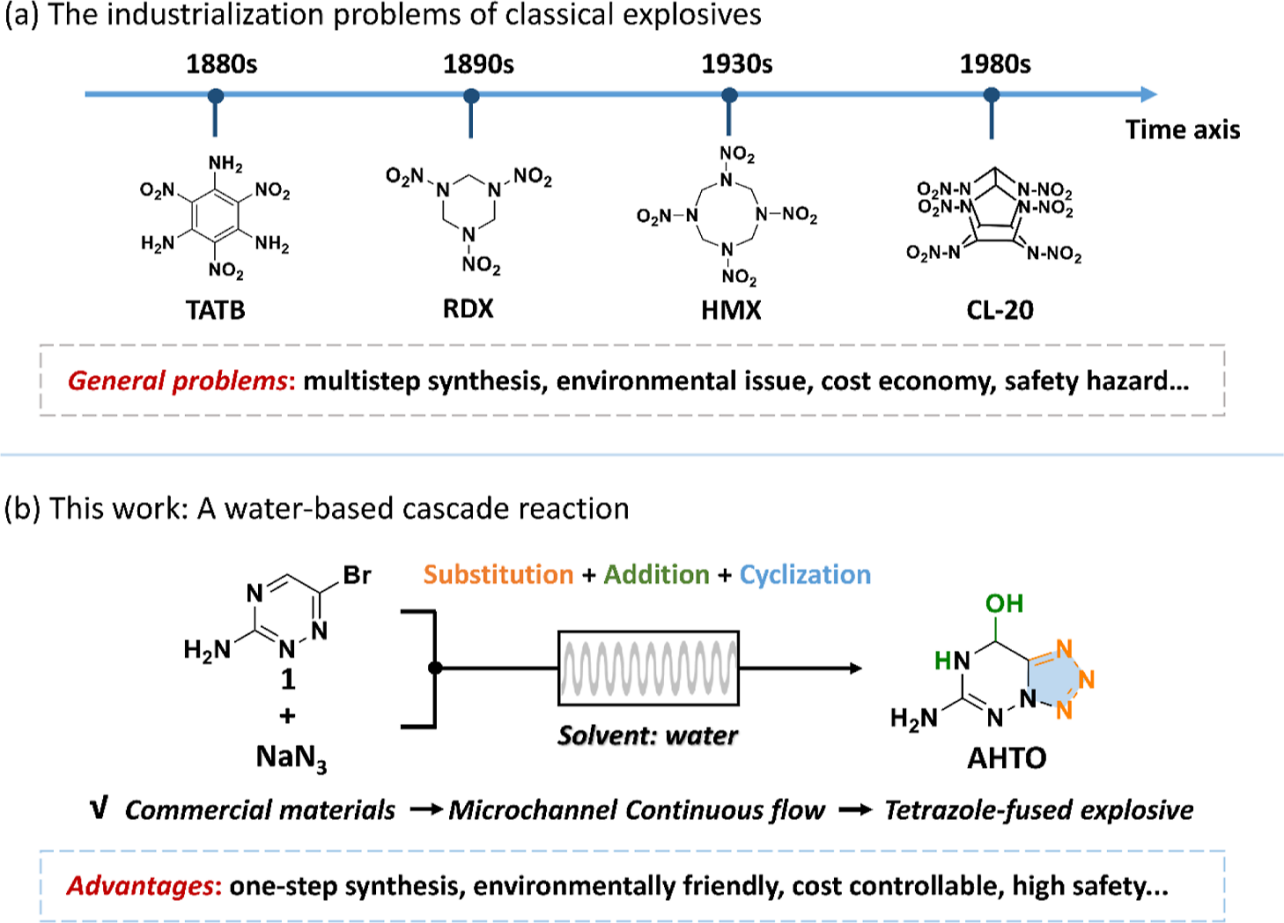
(a) General problems in the industrialization of classical explosives; (b) the water-based cascade reaction in this work.

Cascade reactions are a fascinating branch of organic chemistry.^14,15^ In fact, the savings involved in performing multiple conversions in a single synthetic operation are considerable and can show strong advantages in various aspects (e.g., productivity, resource management, waste treatment, etc.).^16,17^ Thus, the cascade reaction itself fits well with the concept of green chemistry.^18^ Currently, cascade reactions have shown great vigor in the total synthesis of complex natural products.^19^ The heterocyclic structures involved have similar nitrogen-rich backbones and theoretically have the possibility of being derived to the level of energetic compounds.^20,21^ Along with the trend of developing new energetic compounds, the cascade reaction promises to avoid cumbersome traditional steps. However, due to the inherent design difficulty of cascade reactions and the potential exacerbation of sensitive energetic intermediates, this concept has not yet been generalized in the field of energetic materials. It is crucial to find a system that can initiate a cascade and yet ensure a continuous reaction of metastable energetic structures.

Water, a nontoxic, inexpensive, and recyclable solvent, serves as a multifunctional platform for synthesizing energetic compounds, uniquely addressing stability and safety challenges inherent to high-energy reactions.^22,23^ For the construction of energetic compounds, water can both neutralize the risk of instability during azide-related reactions and be a potentially beneficial reagent. The details are as follows: (1) stabilization of energetic reaction systems; (2) promoting some nucleophilic, cyclization, and other reactions; (3) promoting direct crystallization of products to yield high-purity crystals with controlled morphology; and (4) acting as the usual solvent in continuous flow, which is an efficient and green technology to support safe production. Thus, water can be a multifunctional medium as the solvent, stabilizer, reactant, and catalyst. When they are adapted with suitable substrates, it is feasible to develop new energetic compounds via water-based cascade reactions. When integrated with flow chemistry, water acts as both a reaction medium and a heat-transfer fluid, aligning with green chemistry principles for sustainable industrialization.

With the vision of illuminating the efficient green synthesis of energetic materials, this work provides a water-based cascade reaction to obtain 6-amino-7,8-dihydrotetrazolo­[5,1-*f*]­[1,2,4]­triazin-8-ol (**AHTO**), a new tetrazolo-fused energetic compound. As shown in Figure 1b, starting from commercially available 6-bromo-1,2,4-triazin-3-amine (**1**) and NaN_3_, the cascade reaction is carried out using its active C = N bond and the halogen site. Water not only acts as a solvent but also as a nucleophilic reagent and catalyst to drive the addition and cyclization reactions. Moreover, water plays a key role in stabilizing energetic systems so that the cascade reaction can be carried out smoothly. This provides a solution to a long-standing safety issue of the tetrazolo-fused heterocycle, an important nitrogen-rich energetic backbone but whose construction methods generally carry risks that restrict industrialization. And the efficiency can be further enhanced with the addition of microchannel continuous-flow technology, which brings a better mixing effect of starting materials. Thanks to the cascade reaction and continuous-flow technology, the **AHTO** obtained reaches a commercially saleable purity (>98%), a surprising crystallization yield (>90%), and a short preparation time (3 h). Furthermore, a new potential metal-free primary explosive 6-nitroamino-7,8-dihydrotetrazolo­[5,1-*f*]­[1,2,4]­triazin-8-ol (**NHTO**) can be produced by the nitroamino derivatization of **AHTO**. This confirms the potential of **AHTO** for its versatility.

## 2 Results and Discussion

### 2.1 Synthesis and Crystal Structures

Compound **1** underwent the cascade reaction in the NaN_3_/H_2_O solution (Figure 2a). After refluxing for 16 h in the oil bath condition, the crystallization yield of **AHTO** was 62.5%, and the purity was 94.8%. Continuous flow can be used to replace the oil bath conditions (Figure 2b). An aqueous solution of the two reactants (5 mmol/L) is passed into pumps, which pass through the plate reactor and then through a pressure-variable valve, finally picking up an aqueous solution of **AHTO**. Then, **AHTO** is separated by sedimentation. The maximum liquid holding capacity of the monolithic microreactor is 10 mL. The reaction time can be shortened to 3 h while achieving the high crystallization yield of 90.8% and the excellent purity of 98.1%. The detailed system configuration can be found in Figure S2. **AHTO** can be further derivatized to obtain **NHTO** in the crystallization yield of 83.4% (Figure 3). Both **AHTO** and **NHTO** are obtained as crystals directly (Figure S1). The HPLC trace of **AHTO** obtained by the oil bath and the continuous-flow conditions can be found in Figure S13.

**Figure 2:**
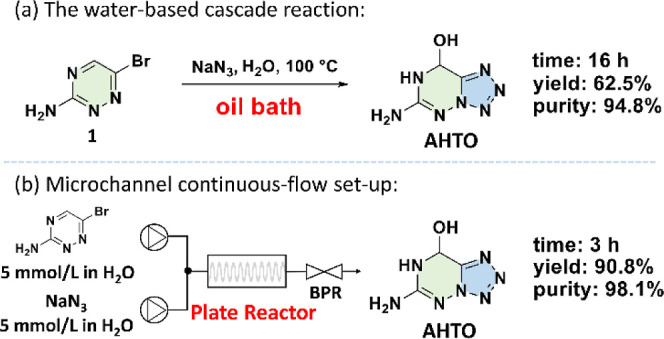
(a) The water-mediated cascade under conventional conditions (both are crystallization yields); (b) the microchannel continuous-flow setup (BPR = Back Pressure Regulator).

**Figure 3:**
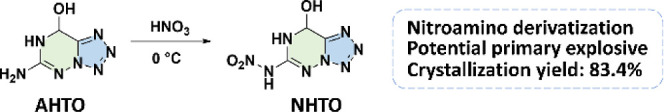
**AHTO** derivatization.

### 2.2 Reaction Mechanism

Water played a variety of roles in driving the cascade reaction. The proposed cascade mechanism is shown in Figure 4. In the first step, along with the departure of the halogen, the azide anion underwent a nucleophilic attack, contributing to the substitution reaction. When using acetonitrile as the solvent, substrate 1 failed to react with sodium azide, indicating that this substitution reaction was more readily conducted via an SN1 mechanism and constituted the first step in the cascade.^24^ In the second step, the azide group acted as an electron-withdrawing group (EWG) forming a withdrawing-induced effect on the C=N double bond.^25^ Water acted as a nucleophilic reagent for the addition reaction. In the third stage, the protonation environment catalyzed the cyclization reaction.^26^ And the heating condition was also conducive to the cyclization reaction.^27^ Water provided a protonation environment as a catalyst for the cyclization.

**Figure 4:**
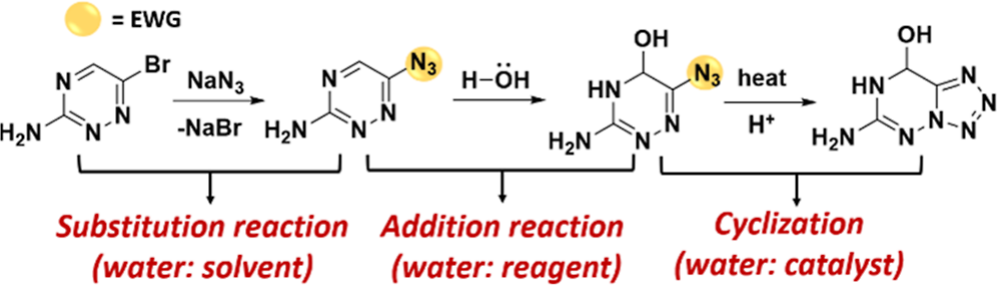
Proposed mechanism.

### 2.3 Single-Crystal X-ray Diffraction

The structures of **AHTO** and **NHTO** were characterized by multinuclear NMR, infrared spectroscopy, and elemental analysis. In particular, the crystals of both **AHTO** and **NHTO** can be obtained directly by the respective synthesis steps, as detailed in the Supporting Information. The crystal of **AHTO** belongs to the *C*2/*c* space group (Z = 8) and the monocyclic crystal system (Figure 5a). The cell volume is 1227.4(2) Å^3^. The corresponding crystal density is 1.679 g·cm^–3^ at 170 K. Due to the presence of the nonaromatic fragment, the skeleton contains a dihedral angle of 14.1° (Figure 5b). As shown in Figure 5c, **AHTO** has mixed stacking. The hydrogen-bonding region is mainly between the nitrogen-rich backbone and the hydrogen atoms. Intermolecular hydrogen-bonding system is expected to affect the stacking. Therefore, it is possible to modulate the hydrogen-bonding regions or even the stacking arrangement by further modification of the substituent groups.

**Figure 5:**
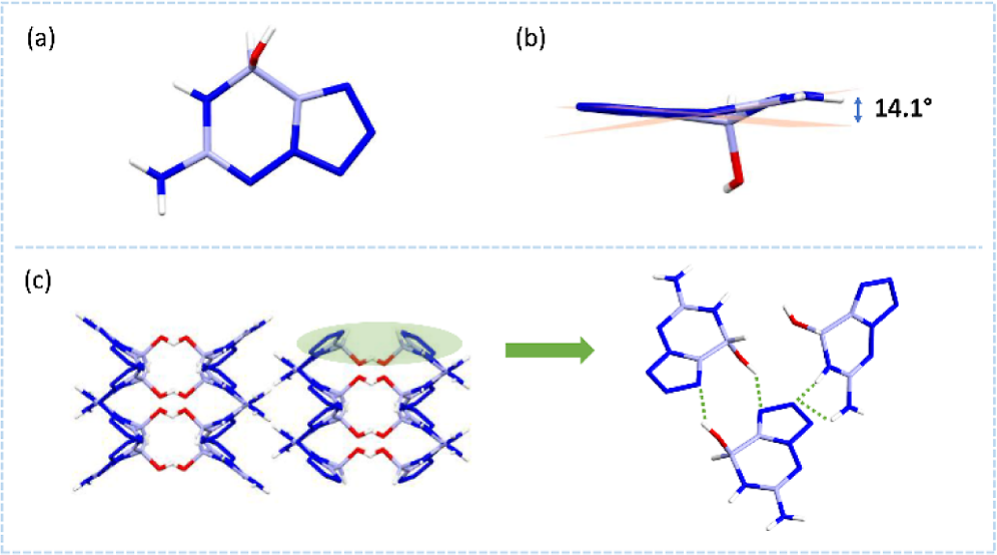
(a) The single-crystal X-ray structures of **AHTO**. (b) The backbone planarity of **AHTO**. (c) The packing diagram and hydrogen-bonding interactions of **AHTO**.

The crystal of **NHTO** belongs to the *Pbca* space group (Z = 8) and the orthorhombic crystal system (Figure 6a). The cell volume is 1422.79(8) Å^3^. The corresponding crystal density is 1.869 g·cm^–3^ at 170 K. More crystal data can be found in Table S1 in the Supporting Information. When the amino group is modified to a nitroamino group, the dihedral angle (10.7°) is subsequently reduced (Figure 6b). As shown in Figure 6c, **NHTO** also has a wave-like stacking. The nitroamino group not only has better energy but also possesses proton donors and acceptors. This provides more options for the formation of dense intermolecular hydrogen bonds with backbones or other groups, promoting more regular stacking and superior overall performance. To further investigate the structure–property relationships, electrostatic potential (ESP) and noncovalent interaction plots (NCI) models are provided (Figure S6 in the Supporting Information).

**Figure 6:**
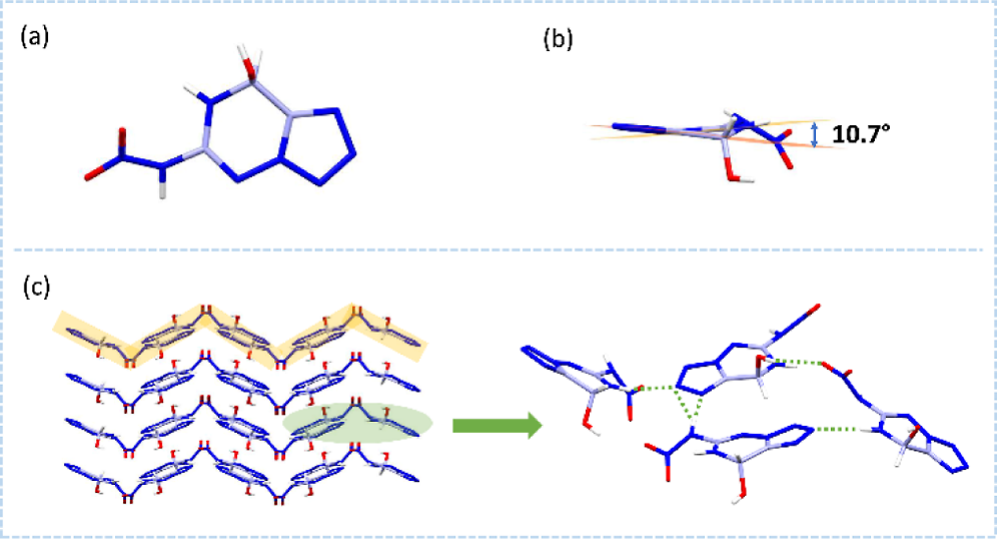
(a) The single-crystal X-ray structure of **NHTO**. (b) The backbone planarity of **NHTO**. (c) The packing diagram and hydrogen-bonding interactions of **NHTO**.

### 2.4 Properties and Application Value

Performance testing has shown that fully functionalized fused rings **AHTO** and **NHTO** each have potential application directions. They have been evaluated by their energy and safety properties. All practical tests used samples that had passed elemental analysis. And calculated properties were obtained using the Gaussian 09 package and EXPLO5 software (version 6.05).^28^ Energy properties include density, heat of formation enthalpy (ΔH_f_), detonation pressure (P), and detonation velocity (D). The classical explosives 2,4,6-trinitrotoluene (TNT), RDX, and lead azide (LA) are also shown in Table 1.^29^ The density was measured at 25 °C by using a gas pycnometer.^30^ The heat of formation (ΔH_f_) was determined based on the isodesmic reaction (detailed in the Supporting Information) by using the Gaussian 09 package.^31^ The detonation pressure (P) and detonation velocity (D) were calculated based on experimental densities and HOF by EXPLO5.^32^ Safety properties include thermal decomposition temperature, impact sensitivity (IS), and friction sensitivity (FS). Differential scanning calorimetry (DSC) analysis was carried out at a heating rate of 5 °C min^–1^.^33^ The TGA-DSC curve shows that **AHTO** is accompanied by a stepwise decomposition process, which may be related to its nonaromatic structure (Figure S9). The introduction of the nitroamino group affected the thermal decomposition temperature of **NHTO** (118 °C), which is lower than that of the three classic explosives listed in Table 1.

**Table 1:** Physiochemical Properties of **AHTO**, **NHTO**, Classic Insensitive Explosive TNT, Secondary Explosive RDX, and Metal Primary Explosive LA

compound	T_*dec*_ ^a^/°C	d^b^/g·cm^–3^	ΔH_f_ ^c^/kJ mol^–1^	v^d^/m·s^–1^	P^e^/GPa	IS^f^/J	FS^g^/N
**AHTO**	158	1.650	298.1	7776	20.9	30	300
**NHTO**	118	1.834	376.9	8753	30.9	<1	20
**TNT** ^h^	295	1.65	–67.0	6881	19.5	15	353
**RDX** ^i^	204	1.80	70.3	8795	34.9	7.4	120
**LA** ^j^	315	4.80	450.1	5920	33.8	2.5–4	0.1–1

a Thermal decomposition temperature.

b Measured density of anhydrous compounds using a gas pycnometer.

c Calculated heat of formation.

d Calculated detonation velocity.

e Calculated detonation pressure.

f Impact sensitivity.

g Friction sensitivity.

h Ref 32.

i Ref 30.

j Ref 2.

Impact sensitivity (IS) and friction sensitivity (FS) were measured based on the standard BAM methods.^34^ The results show that **AHTO** performs similarly to TNT, a widely used secondary explosive, and has a higher detonation performance (7776 m s^–1^, 20.9 GPa). The explosive compound **NHTO** is a derivative of **AHTO**. The density of **NHTO** is 1.834 g cm^–3^, exceeding that of RDX (1.80 g cm^–3^). It is worth noting that **NHTO** is very sensitive (IS < 1 J). Therefore, **NHTO** has energy properties similar to those of the classic explosive RDX and has similar high sensitivities as the classic primary explosive lead azide (LA). As shown in Figure 7, a hot needle test was provided. When **NHTO** placed on lead plates was ignited by the hot needle, it showed a short deflagration-to-detonation transition time of **NHTO**. The hot needle test confirmed the priming performance of **NHTO**.

**Figure 7:**
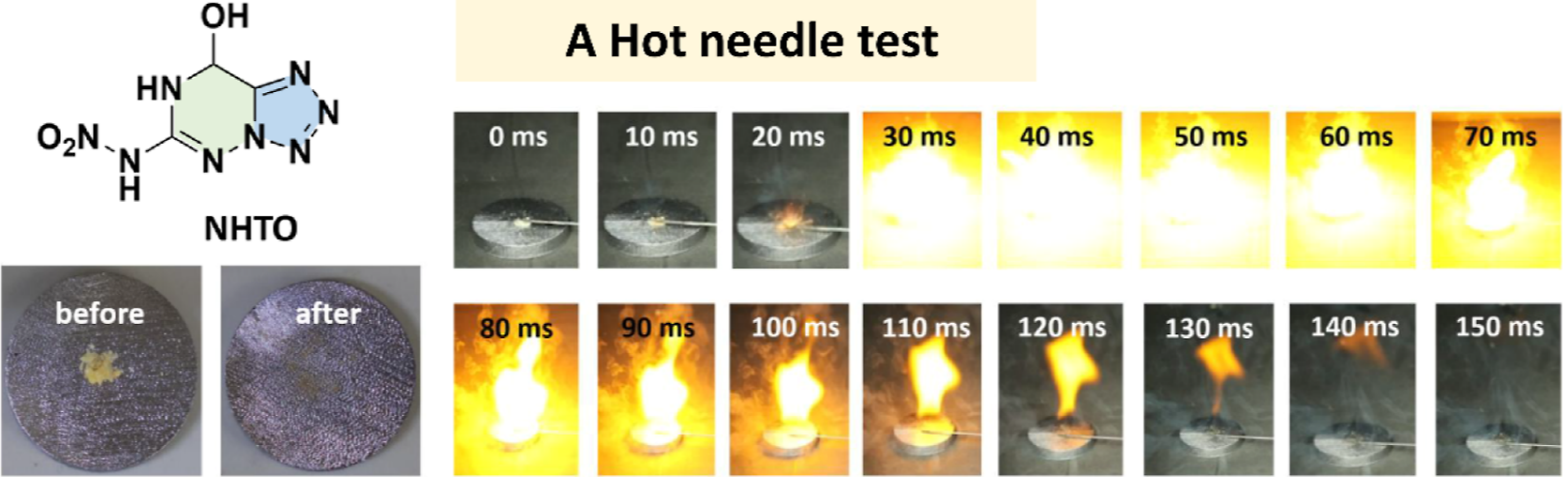
Hot needle test of **NHTO**.

Demonstrated stability during storage is a fundamental prerequisite for considering any explosive material for potential application.^35,36^ To further clarify the stability of the resulting energetic compounds, especially the uncertain effects of hydroxyl, N–H, and other activities, the air stability test was done for both **AHTO** and **NHTO**. Both maintained good stability over three months in a conventional storage environment and retained the same color as when freshly prepared. This has been well confirmed by IR spectroscopy (Figure 8). It is an important basis to support subsequent scale-up in volume preparation and application. Under current continuous-flow conditions, the cost of **AHTO** can be controlled at $500 per kg.

**Figure 8:**
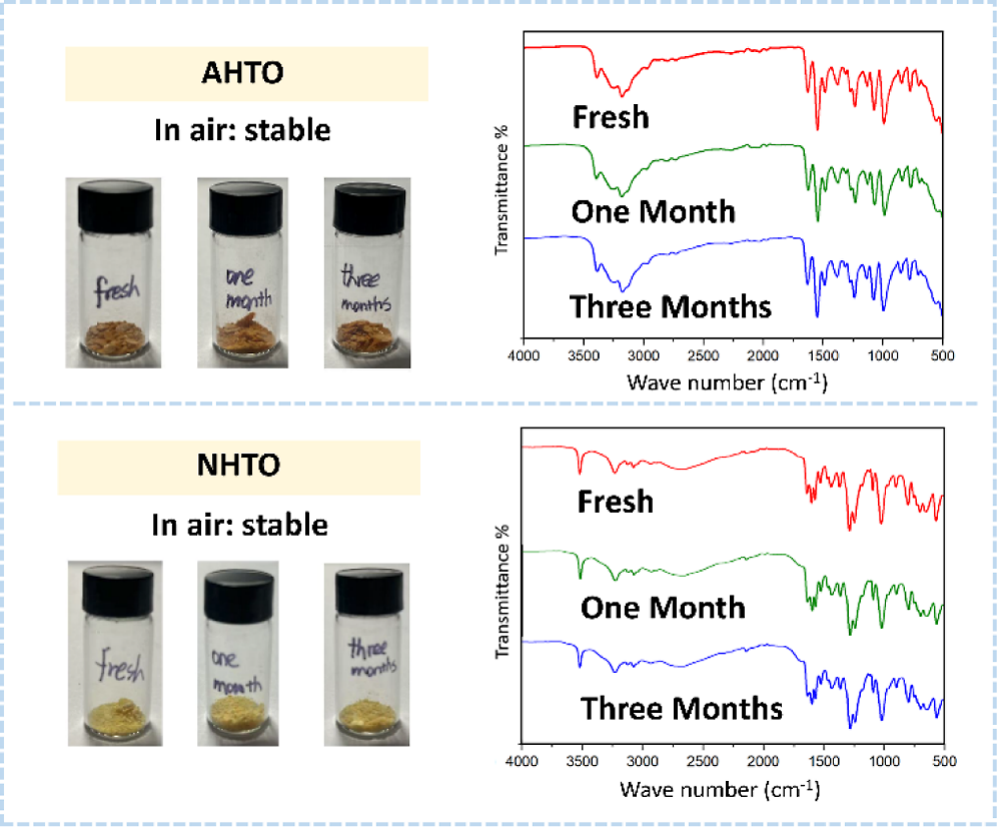
Air stability of **AHTO** and **NHTO**.

## 3 Conclusions

In summary, this work presents a water-based cascade reaction to develop new tetrazolo-fused heterocycles **AHTO** and **NHTO**. Both can be obtained directly in crystals. As a multifunctional medium, water acts simultaneously as a solvent, nucleophile, catalyst, and stabilizer. The cascade integrates dangerous azides going through substitution, addition, cyclization into a single safe operation to obtain the tetrazolo-fused heterocycle **AHTO**. When further introducing the microchannel continuous-flow technology, the cascade reaction time was only 3 h, achieving a 90.8% crystallization yield and a 98.1% HPLC purity. Both **AHTO** and its derivative product **NHTO** have passed IR, NMR, elemental analysis, and X-ray diffraction. **AHTO** is expected to be a “blunt” explosive like TNT. With a short deflagration-to-detonation transition time, **NHTO** is a potential metal-free explosive. The water-based cascade provides a reference for balancing economic, safety, and environmental considerations. The continuous-flow technology establishes a pathway for future scale-up.

## Supplementary Material



## Notes

Experimental section, X-ray crystallography, theoretical calculations, NMR spectra and TGA-DSC curve of **AHTO** and **NHTO**, and HPLC trace of **AHTO** (PDF).
