# Supplementary material for: A Water-Based and Continuous-Flow-Usable Cascade: Sustainable Synthesis of Tetrazolo-Fused Heterocycles
Source: JACS Au. 2025 Oct 24;5(11):5590–5. doi: 10.1021/jacsau.5c01089 (PMC12648318; doi:10.1021/jacsau.5c01089)
Supplement: Supplementary file 1 [file au5c01089_si_001.pdf]

Supporting Information

A Water-Based and Continuous-Flow-Usable Cascade: Sustainable Synthesis of Tetrazolo-Fused Heterocycles

Yaxi Wang [a], Suxian Fu [a], Deshu Kong [a], Lu Hu [a*] Siping Pang [a*] and Jean'ne M. Shreeve [b*]

[a] *School of Materials Science & Engineering, Beijing Institute of Technology, Beijing 100081, China*

[b] *Department of Chemistry, University of Idaho, Moscow, ID 83844-2343, USA*

E-mail: Lu Hu (lhu@bit.edu.cn); Siping Pang (pangsp@bit.edu.cn), Jean'ne M. Shreeve (jshreeve@uidaho.edu)

Contexts

1. Experimental section 1

2. X-ray Crystallography 3

3. Theoretical calculations 4

4. NMR spectra and TGA-DSC curve of **AHTO** and **NHTO** 6

5. HPLC trace of **AHTO** 9

References 9

1. Experimental section

Caution! Although we have not experienced any difficulties in preparing and handling these new energetic materials, proper protective precautions must be used. All compounds should be handled with care using the best safety practices.

General Methods: All reagents were obtained from TCL and were used as supplied. In the continuous-flow setups, two JINGJIN JJRZ-10004F pumps were used: 5 mmol/L compound 6-bromo-1,2,4-triazin-3-amine and NaN_3. The continuous-flow instrument consisted of three HZSS 0010WR-C276 reactors. The reaction temperature was controlled by a refrigerated-heated circulator (Labtemp DCC14). A Bruker AVANCE 400 nuclear magnetic resonance spectrometer operating at 400, and 101 MHz was used to collect ^1H and ^{13}C spectra, respectively. DMSO-d_6 was employed as solvent and locking solvent. Chemical shifts are given relative to Me_4Si for ^1H and ^{13}C spectra. Thermal decomposition (onset) points were measured by a differential scanning calorimeter (TA Instruments Co., model Q2000) at a scan rate of 5 °C min^{-1}. Densities were determined at room temperature by a Micromeritics AccuPyc 1340 gas pycnometer. IR spectra were recorded on a FT-IR spectrometer (Thermo Nicolet AVATAR 370) as thin films using KBr plates. The impact and friction sensitivities were obtained by using a standard BAM Fallhammer and a BAM friction tester. Elemental analyses (CHNS) were performed on a Vario Micro cube Elementar Analyser. High performance liquid chromatography (HPLC) was performed on a ThermoFisher-

UltiMate 3000.

6-bromo-1,2,4-triazin-3-amine (1) was obtained commercially.

6-amino-7,8-dihydrotetrazolo[5,1-*f*][1,2,4]triazin-8-ol (AHTO)

Method A: Compound 6-bromo-1,2,4-triazin-3-amine (875.0 mg, 5 mmol) was mixed with water (18.0 mL) at room temperature. Then, NaN_3 (360.0 mg, 5.5 mmol) was added to it in batches, and transferred to reflux environment for 16 h. The cloudy system was gradually transformed into a clear brown solution. Upon stopping the heating, a large quantity of crystals precipitated rapidly. After filtration and drying, the crystalline **AHTO** was obtained (484.2 mg, crystallization yield = 62.5%). ^{1}H NMR ($[D_6]$DMSO, 400 MHz): δ 6.21 (s, 2H, NH2), 6.26 (dd, 1H, C-H), 7.10 (d, 1H, N-H), 8.13 (d, 1H, O-H); ^{13}C NMR ($[D_6]$DMSO, 101 MHz): 67.5, 140.0, 152.8 ppm. IR (KBr pellet): 3397, 3188, 1651, 1569, 1509, 1404, 1334, 1298, 1265, 1153, 1101, 1030, 1018, 888, 804, 734, 701, 485 cm^{-1}; elemental analysis ($C_3H_5N_7O$, 155.12): calcd C 23.23, H 3.25, N 63.21; found C 22.98, H 3.00, N 62.87.

Method B (continuous flow): After weighing compound 6-bromo-1,2,4-triazin-3-amine (175.0 mg, 1.0 mmol) and sodium azide (65.0 mg, 1.0 mmol), aqueous solutions were prepared at concentrations of 5 mmol/L. The two solutions were passed into pump 1 (P1) and pump (P2), and the flow rate was set to 0.08 mL/min in both cases. All three plate reactors were operated in a refrigerated-heated circulator with the temperature at 100 °C. After passing through the Back Pressure Regulator (BPR), the hot aqueous solution of the **AHTO** product is collected. Upon cooling, crystallization of **AHTO** occurred (140.1 mg, crystallization yield = 90.8%).

6-nitroamino-7,8-dihydrotetrazolo[5,1-*f*][1,2,4]triazin-8-ol (NHTO)

To fuming nitric acid (4.0 mL) was added **AHTO** (155.1 g, 1.0 mmol) in small portions with stirring at 0 °C. The reaction mixture was stirred for 40 minutes at 0 °C. The reaction could be detected by TLC. To the solution, ice (5.0 g) was added to obtain a clarified orange solution. The solution was extracted with ethyl acetate (3 x 20 mL), and washed with a little cold acetonitrile (3 mL) to give 166.9 mg (crystallization yield = 83.4 %) yellow crystalline **NHTO**. ^{1}H NMR ($[D_6]$DMSO, 400 MHz): δ 6.59 (d, 1H, C-H), 10.26 (d, 1H, N-H); ^{13}C NMR ($[D_6]$DMSO, 101 MHz): 68.7, 141.3, 145.4 ppm; IR (KBr pellet): 3519, 3229, 3074, 2655, 1634, 1596, 1570, 1525, 1434, 1363, 1280, 1241, 1176, 1092, 1022, 897, 795, 721, 701, 648, 564, 506, 417 cm^{-1}; elemental analysis ($C_3H_4N_8O_3$, 200.12): calcd C 18.01, H 2.01, N 55.99; found C 17.59, H 1.99, N 56.03.

Products: The crystallization products of **AHTO** and **NHTO** are shown in Figure S1.

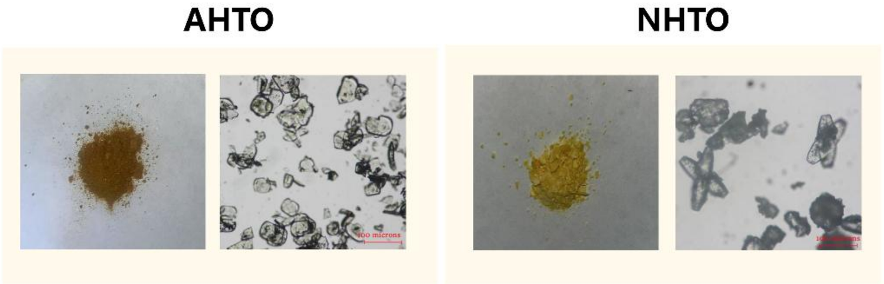

Figure S1. The crystallization products (scale = 100 microns) of **AHTO** and **NHTO**.

System configuration: As shown in Figure S2, the two samples were introduced via pumps, then entered the plate reactor for mixing. The refrigerated-heated circulator unit should be preheated half an hour before the reaction and brought to a temperature of 100 ℃. Following a three-hour reaction, the hot **AHTO** solution was collected and allowed to settle, yielding **AHTO** single-crystal

product after cooling down. Depending on the reaction conditions, a back pressure regulator can be connected at the outlet.

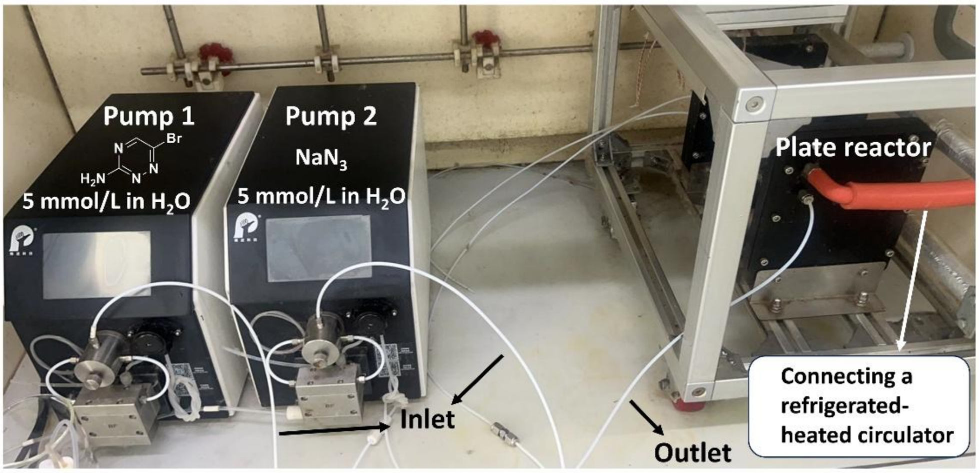

Figure S2. System configuration.

2. X-ray Crystallography

Crystal Structure Analysis: The X-ray diffraction data were obtained on a Bruker D8 Venture PHOTON II CPAD diffractometer equipped with a Cu/Mo Kα INCOATEC ImuS micro-focus source. Indexing was performed using Apex3.[1] Data integration and reduction were performed using Saint.[2] Absorption correction was performed by a multi-scan method implemented in SADABS.[3] Space groups were determined using XPREP implemented in APEX3[4]. The structure was solved using SHELXT and refined using SHELXL-2018[5-7](full-matrix least-squares on F^2) within OLEX2 interface program. All non-hydrogen atoms were refined anisotropically. All hydrogen atoms were placed in geometrically calculated positions and were included in the refinement process using riding models with isotropic thermal parameters. Crystal data is shown in Table S1. Figure S3-S4 are the crystal structures for **AHTO** and **NHTO**.

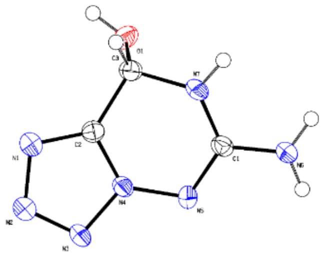

Figure S3. Single-crystal X-ray structure of **AHTO** (50% probability level).

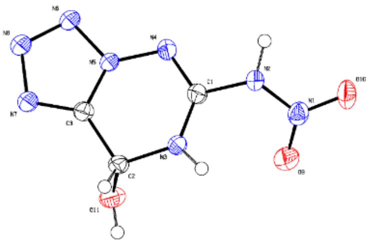

Figure S4. Single-crystal X-ray structure of **NHTO** (50% probability level).

Table S1. Crystal Data

	AHTO	NHTO
CCDC No.	2447514	2447517
Formula	$C_3H_5N_7O$	$C_3H_4N_8O_3$
$D_{calc.}$/ g·cm^{-3}	1.679	1.869
μ/mm^{-1}	0.135	1.444
Formula Weight	155.14	200.14
Color	Brown	yellow
Shape	block	block
Size/mm^3	0.12×0.06×0.05	0.11×0.04×0.02
T/K	170	173
Crystal System	monoclinic	orthorhombic
Space Group	C2/c	Pbca
a/Å	22.295(3)	11.7201(4)
b/Å	4.0354(5)	8.5202(3)
c/Å	13.7569(15)	14.2482(4)
α/°	90	90
β/°	97.383(7)	90
γ/°	90	90
V/Å^3	1227.4(2)	1422.79(8)
Z	8	8
Radiation type	Mo Kα	Cu Kα
$2\Theta_{min}$/°	5.972	12.424
$2\Theta_{max}$/°	52.762	133.128
Measured Refl's.	5981	2276
Ind't Refl's	1242	1222
R_{int}	0.0977	0.0273
R_{sigma}	0.0797	0.0341
Parameters	113	136
Restraints	3	1
Index ranges	-27≤ h ≤ 26, -5 ≤ k ≤ 5, -17 ≤ l ≤ 17	0 ≤ h ≤ 13, -9 ≤ k ≤ 9, -16 ≤ l ≤ 0
Largest Peak/e Å^{-3}	0.33	0.19
Deepest Hole/e Å^{-3}	-0.33	-0.30
GooF	1.086	1.138
wR_2 (all data)	0.1225	0.1076
wR_2	0.0992	0.1025
R_1 (all data)	0.1038	0.0499
R_1	0.0530	0.0423

3. Theoretical calculations

Calculations: The calculations of the heats of formation were carried out using Gaussian 09 (Revision 6.05) suite of programs. All the compounds were determined using isodesmic reactions (Fig. S3). The geometric optimization and frequency analyses of the structures were calculated using B3LYP/6-31+G** level. The gas phase enthalpy of formation was computed and the enthalpy of reaction was obtained by combining the MP2/6-311++G** energy difference for the reactions,

the scaled zero- point energy (ZPE), values of thermal correction (HT), and other thermal factors. All the optimized structures were characterized to be true local energy minima on the potential energy surface without imaginary frequencies[8].

3.1 Heats of formation

The isodesmic reactions (Figure S5) for **AHTO** and **NHTO** all follow by the actual crystal structures.

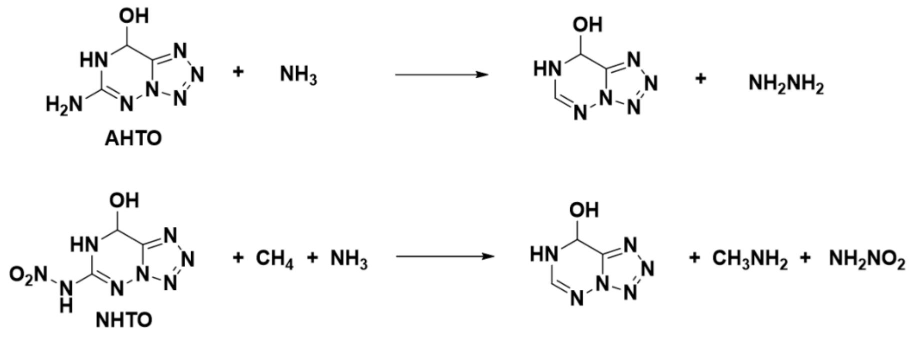

Figure S5. Isodesmic reactions for compounds **AHTO** and **NHTO**.

3.2 ESP and NCI analysis

To further establish the sensitivity-structure relationship, an electrostatic potential (ESP) analysis derived from the crystal structure was applied. Recent research has demonstrated a strong correlation between ESPs and impact sensitivities (Figure S5a).[9] The ESP was analyzed based on the B3LYP/6-311G(d,p) method. The positive and negative potentials are shown in red and blue. The maxima and minima of ESP are expressed as orange and cyan spheres, respectively. Compared to **AHTO**, the nitroamino derivatization in **NHTO** leads to a more pronounced positive potential on its hydrogen atoms, indicating enhanced reactivity and sensitivity. Under external mechanical stimuli (such as impact or friction), these highly positive hydrogens can strongly interact with adjacent negative moieties, promoting bond cleavage and the subsequent release of energy. This electronic structure feature provides an explanation for the markedly higher mechanical sensitivities of **NHTO** (< 1 J, 20 N) compared to **AHTO** (30 J, 300 N).

Noncovalent interaction plots (NCI) are widely used to study hydrogen bonds, van der Waals interactions, and repulsive steric clashes (Figure S5b).[10] The surfaces are colored in a blue-green-red scale indicating strong attractive interactions, weak interactions, and strong nonbonded overlap, respectively. As shown in Figure S5b, two molecules from the interlayer in **AHTO** and **NHTO** were selected. The results show that there is a continuous π-π stacking between **AHTO**-**AHTO**, which is well buffered against external stimuli. For **NHTO**, π-π stacking only exists between the backbones and is discontinuous. Moreover, the nitroamino groups within **NHTO** exhibit no overlapping regions, lacking the necessary condition for π-π interactions. It is one of the reasons for the high sensitivities of **NHTO**.

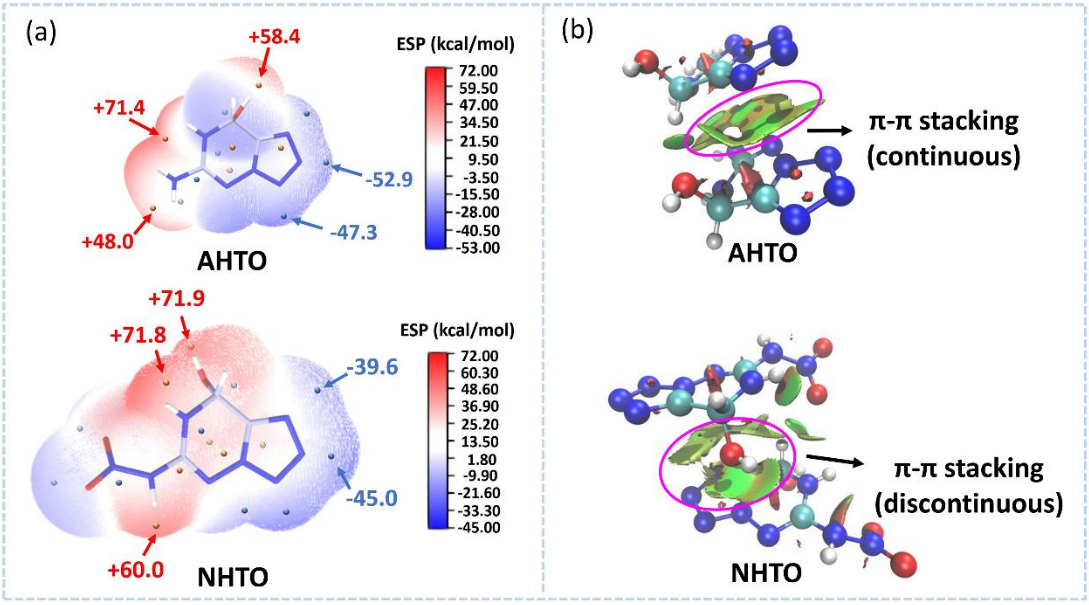

Figure S6. (a) ESP plots of **AHTO** and **NHTO**; (b) NCI plots of **AHTO** and **NHTO**.

4. NMR spectra and TGA-DSC curve of AHTO and NHTO

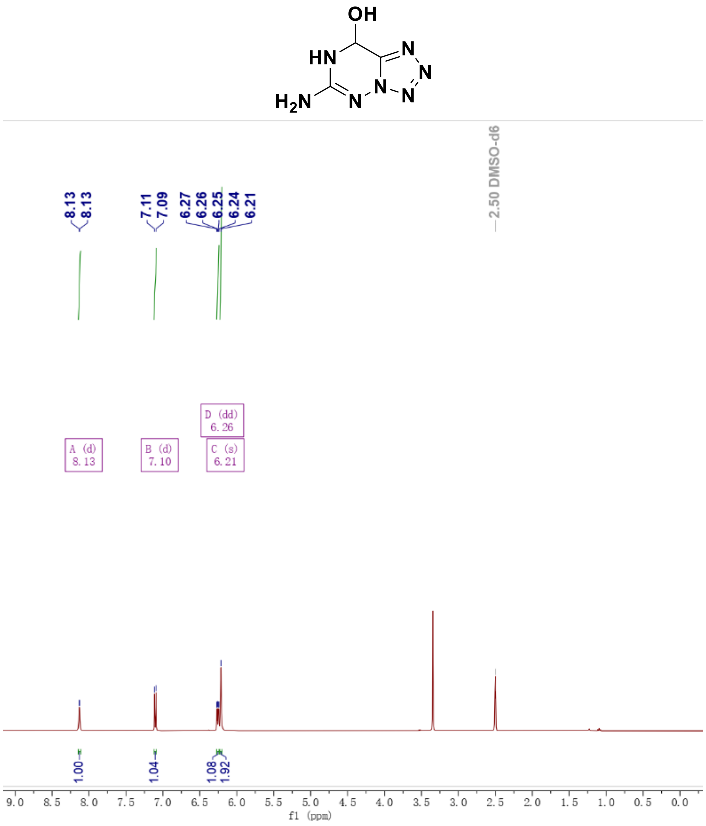

Figure S7. ^{1}H-NMR spectrum of **AHTO** in d_6-DMSO.

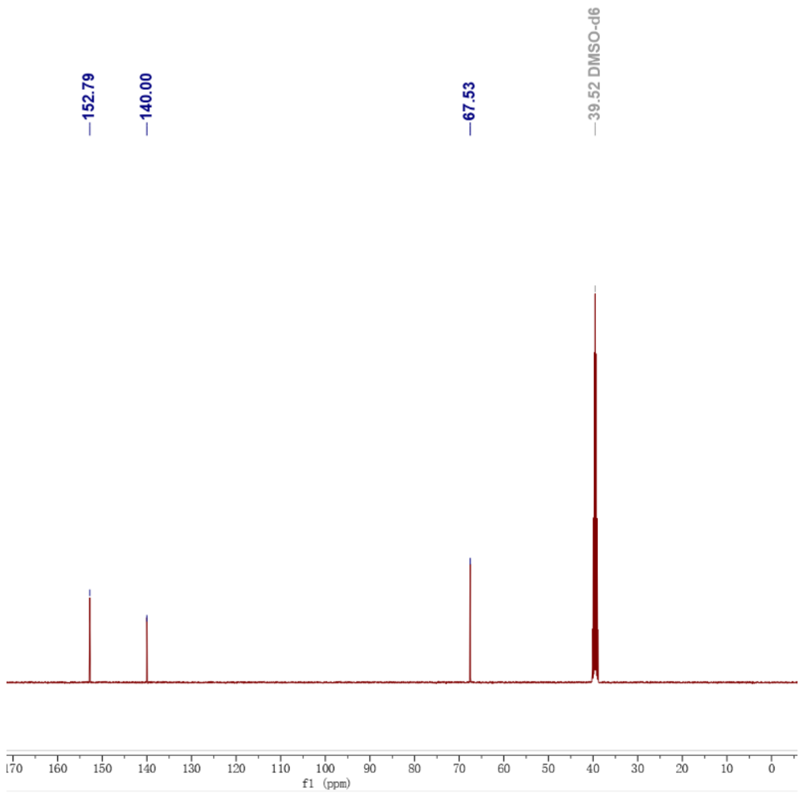

Figure S8. ^{13}C-NMR spectrum of **AHTO** in d_6-DMSO.

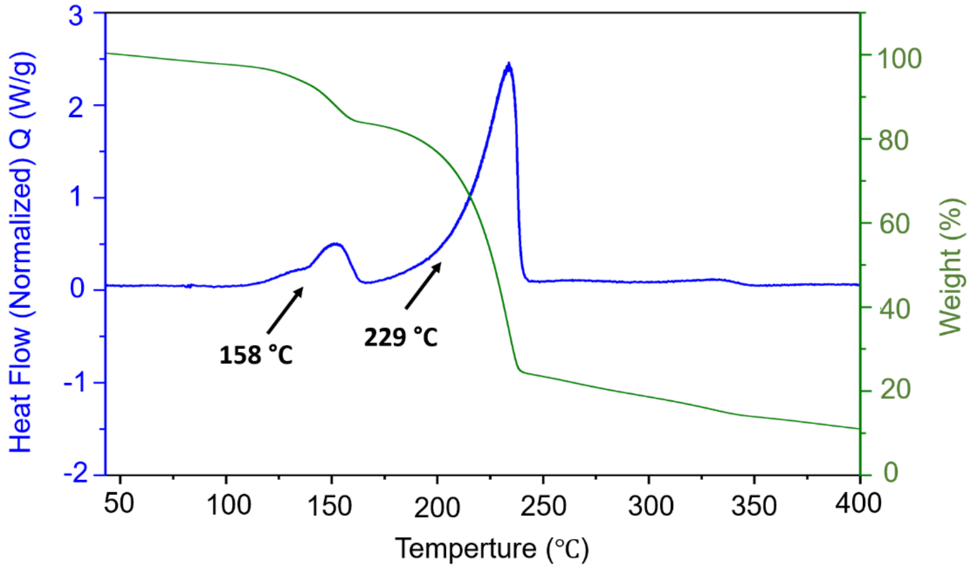

Figure S9. TGA-DSC curve of **AHTO**.

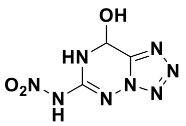

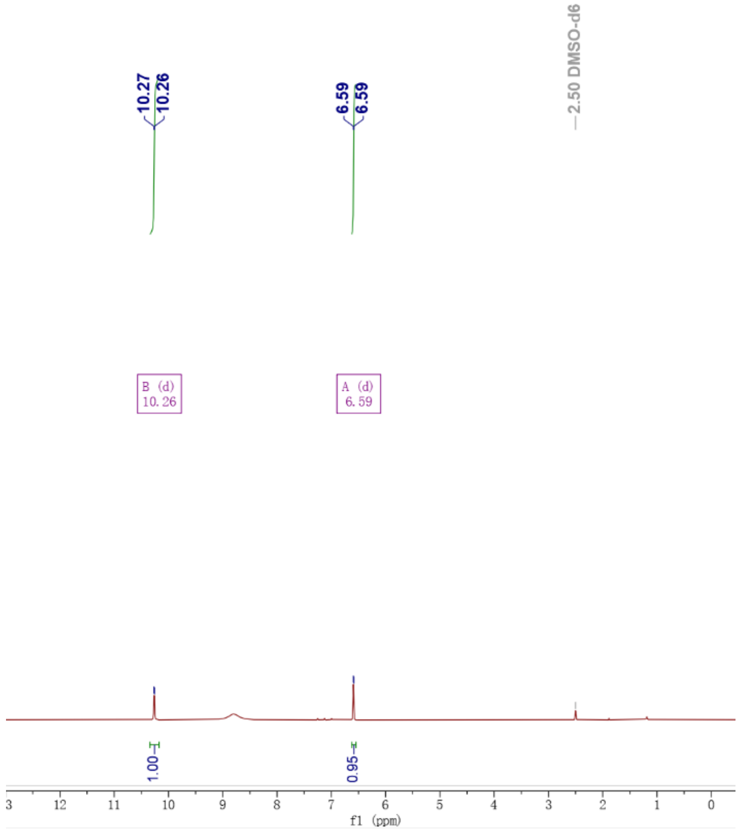

Figure S10. ^{1}H-NMR spectrum of **NHTO** in d_6-DMSO.

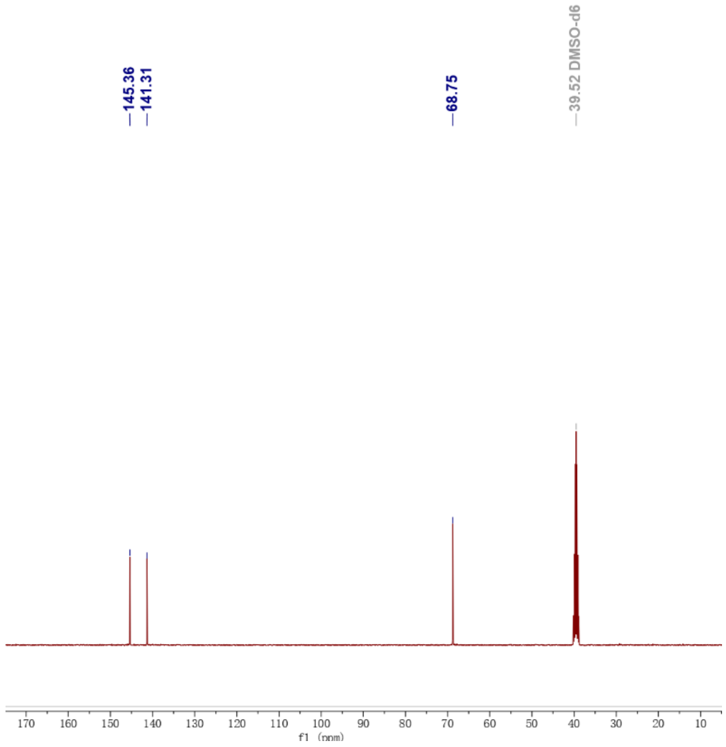

Figure S11. ^{13}C-NMR spectrum of **NHTO** in d_6-DMSO.

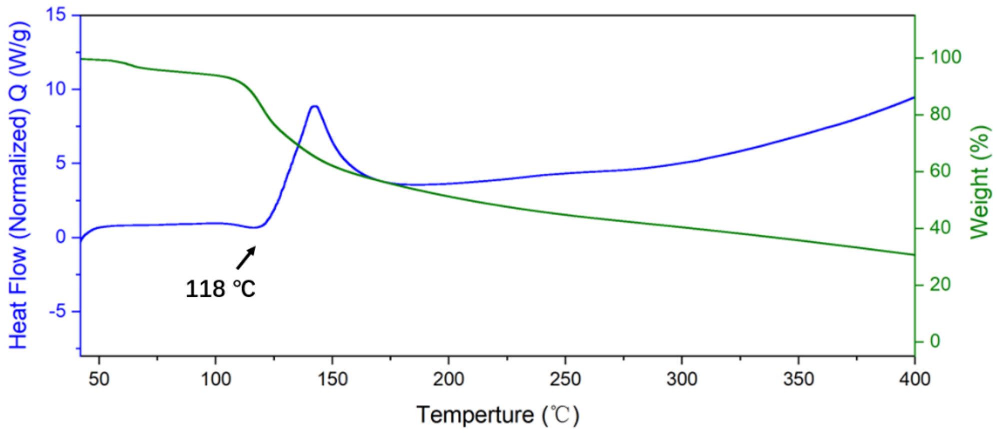

Figure S12. TGA-DSC curve of **NHTO**.

5. HPLC trace of AHTO

The HPLC (acetonitrile: methol = 1:1) showed that the purity of the product **AHTO** obtained in the oil bath was up to 94.5% (Figure S13a). When replaced with the continuous flow, HPLC shows a further increase in product purity to 98.1% (Figure S13b).

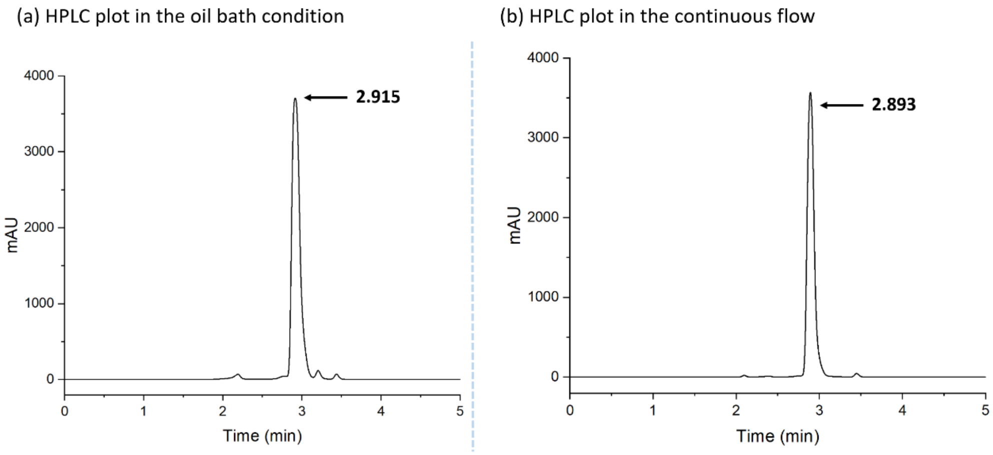

Figure S13. (a and b) The HPLC of **AHTO** obtained by the oil bath and the continuous flow, respectively.

References

(1) Bruker (2018). APEX3 (Version 2017.3). Bruker AXS Inc., Madison, Wisconsin, USA.
(2) Bruker (2018) SAINT V8.35A. Data Reduction Software.
(3) Sheldrick, G. M. (1996). SADABS. Program for Empirical Absorption Correction. University of Gottingen, Germany.
(4) Sheldrick, G. M. "SHELXT - Integrated space-group and crystal structure determination" *Acta Cryst*. **2015**, *A71*, 3-8.
(5) Sheldrick, G.M. *Acta Cryst*. **1990**, *A46*, 467-473.
(6) Sheldrick, G. M. *Acta Cryst*. **2008**, *A64*, 112-122.
(7) G.M. Sheldrick "Crystal structure refinement with SHELXL", *Acta Cryst*. **2015**, *C71*, 3-

8.
(8) Frisch, M. J.; Trucks, G. W.; Schlegel, H. B.; Scuseria, G. E.; Robb, M. A.; Cheeseman, J. R.; Montgomery, J. A.; Vreven, T.; Kudin, K. N.; Burant, J. C.; Millam, J. M.; Iyengar, S. S.; Tomasi, J.; Barone, V.; Mennucci, B.; Cossi, M.; Scalmani, G.; Rega, N.; Petersson, G. A.; Nakatsuji, H.; Hada, M.; Ehara, M.; Toyota, K.; Fukuda, R.; Hasegawa, J.; Ishida, M.; Nakajima, T.; Honda, Y.; Kitao, O.; Nakai, H.; Klene, M.; Li, X.; Knox, J. E.; Hratchian, H. P.; Cross, J. B.; Bakken, V.; Adamo, C.; Jaramillo, J.; Gomperts, R.; Stratmann, R. E.; Yazyev, O.; Austin, A. J.; Cammi, R.; Pomelli, C.; Ochterski, J. W.; Ayala, P. Y.; Morokuma, K.; Voth, G. A.; Salvador, P.; Dannenberg, J. J.; Zakrzewski, V. G.; Dapprich, S.; Daniels, A. D.; Strain, M. C.; Farkas, O.; Malick, D. K.; Rabuck, A. D.; Raghavachari, K.; Foresman, J. B.; Ortiz, J. V.; Cui, Q.; Baboul, A. G.; Clifford, S.; Cioslowski, J.; Stefanov, B. B.; Liu, G.; Liashenko, A.; Piskorz, P.; Komaromi, I.; Martin, R. L.; Fox, D. J.; Keith, T.; Al-Laham, M. A.; Peng, C. Y.; Nanayakkara, A.; Challacombe, M.; Gill, P. M. W.; Johnson, B.; Chen, W.; Wong, M. W.; Gonzalez, C.; Pople, J. A, Gaussian 09, revision D.01; Gaussian, Inc.: Wallingford, CT, **2009**.
(9) Lu, T.; Chen, F. Multiwfn: A multifunctional wavefunction analyzer. *J. Comput. Chem.* **2012**, *33* (5), 580-592.
(10) Zhang, J.; Feng, Y.; Staples, R. J.; Zhang, J.; Shreeve, J. M. Taming nitroformate through encapsulation with nitrogen-rich hydrogen-bonded organic frameworks. *Nat. Commun.* **2021**, *12*, 2146-2152.
